# Functional characterization and safety evaluation of an airway commensal *Staphylococcus epidermidis* HK 95

**DOI:** 10.3389/fmicb.2026.1787099

**Published:** 2026-06-17

**Authors:** Shuwen Lei, Jie Zhong, Rong Chen, Kezhuo Chen, Yi Zheng, Yimeng Dang, Shuoshuo Li, Bo Fang, Wenwen Jin, Longjiang Yu

**Affiliations:** 1 Institute of Resource Biology and Biotechnology, College of Life Science and Technology, Huazhong University of Science and Technology, Wuhan, China; 2 Hubei Engineering Research Center for Both Edible and Medicinal Resources, Wuhan, China; 3 Key Laboratory of Molecular Biophysics of the Ministry of Education, College of Life Science and Technology, Huazhong University of Science and Technology, Wuhan, China; 4 Hubei Provincial Hospital of Traditional Chinese Medicine, Wuhan, China

**Keywords:** airway commensal bacteria, airway function, host–microbe interactions, safety assessment, *Staphylococcus epidermidis*

## Abstract

**Introduction:**

The airway epithelium plays a central role in maintaining respiratory homeostasis, and increasing evidence suggests that resident commensal bacteria contribute to airway protection through host–microbe interactions. However, the functional roles of individual airway commensals remain poorly defined. Here, we aimed to isolate and functionally characterize an airway commensal *Staphylococcus epidermidis* strain and to determine its influence on airway function as well as its safety profile.

**Methods:**

An airway commensal *S. epidermidis* strain, designated HK 95, was isolated and characterized using a combination of *in vivo*, *in vitro*, and genomic approaches. Airway functional responses were evaluated in mice and guinea pigs using citric acid–induced acute cough, phenol red expectoration, and bronchoconstriction models. Safety assessments included cytotoxicity, hemolysis, antibiotic susceptibility testing, and evaluation in mice, integrating relative organ weight indices, longitudinal body weight assessment, and hematological profiling. Whole-genome sequencing and bioinformatic annotation were performed to assess taxonomic identity and safety-related genomic features.

**Results:**

Administration of *S. epidermidis* HK 95 significantly attenuated citric acid–induced airway irritation, as evidenced by reduced cough frequency and prolonged response latency in both mice and guinea pigs. In addition, *S. epidermidis* HK 95 enhanced tracheal secretion in mice, indicating improved airway secretory function, and significantly prolonged preconvulsive time in a guinea pig bronchoconstriction model, suggesting a protective effect on airway responsiveness. Comprehensive safety evaluations demonstrated that *S. epidermidis* HK 95 was non-haemolytic, exhibited a non–multidrug-resistant antibiotic susceptibility profile, and well tolerated following administration in mice, with no significant adverse effects or organ abnormalities. Whole-genome sequencing confirmed its taxonomic identity as *S. epidermidis* and did not identify genetic determinants associated with classical staphylococcal toxins or overt pathogenicity.

**Discussion:**

These findings demonstrate that the airway commensal *S. epidermidis* HK 95 exerts protective effects on airway physiological responses while maintaining a favorable safety profile. This study extends current understanding of airway commensal bacteria beyond compositional associations and supports a functional role for *S. epidermidis* in maintaining airway physiological homeostasis. These results provide a foundation for future studies exploring host–microbe interactions in the airway and the potential development of commensal-based airway interventions.

## 1 Introduction

The airway epithelium constitutes the first line of defense against inhaled environmental stimuli and plays a central role in maintaining respiratory homeostasis. Beyond serving as a physical barrier, airway epithelial cells actively participate in immune regulation, mucociliary clearance, and sensory signal transduction, which together shape airway responsiveness and inflammation (Hewitt and Lloyd, 2021; Ye and Bankova, 2022). Dysregulation of these processes is closely associated with airway symptoms such as cough, mucus hypersecretion, and bronchoconstriction, which are common features of a wide range of respiratory disorders (Israel and Reddel, 2017; Stikker et al., 2023; Zhang et al., 2024).

In recent years, increasing attention has been directed toward the airway microbiota as an additional layer of regulation in respiratory homeostasis (Li et al., 2024; Wang et al., 2023). Culture-independent sequencing studies have revealed that the healthy upper and lower airways harbor diverse microbial communities, distinct from those of the gut but enriched in specific commensal taxa (Dickson, 2016; Dickson et al., 2014a; Zelasko et al., 2024). More recent work has further emphasized that airway microbiota–host interactions extend beyond compositional associations and may actively shape airway function and immune homeostasis (Jaeger et al., 2020; Jiang and Zhang, 2025). Among the resident taxa, *Staphylococcus epidermidis* is one of the most prevalent and persistent species detected in the nasal cavity and conducting airways of healthy individuals (Gioula and Exindari, 2026; Ingham et al., 2025).

Traditionally regarded as a benign skin commensal, *S. epidermidis* has increasingly been recognized for its immunomodulatory roles at epithelial surfaces, including the production of antimicrobial peptides, modulation of host innate immune signaling, and competitive exclusion of pathogenic bacteria (Yang et al., 2024; Zheng et al., 2022). While the functional contributions of the airway microbiota to host defense have begun to emerge, the specific roles of individual commensal species in shaping airway function remain poorly defined. Most existing studies have focused on pathogen-driven inflammation or dysbiosis-associated disease states, whereas far less is known about whether resident commensals actively participate in maintaining normal airway function under non-pathological conditions (Natalini et al., 2023; Yan et al., 2022; Zhu et al., 2025). In particular, whether airway commensal bacteria can influence airway irritation, secretion, or bronchial tone—key determinants of airway function—has not been systematically investigated.

In this study, we isolated an airway-derived *S. epidermidis* HK 95, and evaluated its functional effects on airway responses using a combination of *in vivo* and *in vitro* models. We assessed multiple airway functional responses, including airway irritation responses, tracheal secretion and bronchoconstriction. Considering the potential use of commensals in airway-directed interventions, we further conducted comprehensive phenotypic and genomic safety assessments. By integrating functional characterization with safety evaluation, this work aims to provide insight into the potential airway-protective roles of *S. epidermidis* and to expand current understanding of host–microbe interactions in the respiratory tract.

## 2 Materials and methods

### 2.1 Bacterial strains and basic characterization

From multiple microbial isolates obtained from human nasal swab samples, *S. epidermidis* HK 95 was selected as the representative strain. *D. pigrum* was included as a health-associated nasal commensal comparator where indicated.

*S. epidermidis* HK 95 was routinely cultured on TSA plates or in TSB at 37 °C under aerobic conditions, following standard procedures for coagulase-negative staphylococci (Chiera et al., 2023). *D. pigrum* was cultured on blood agar plates at 37 °C under 5% CO₂, as previously described for upper respiratory tract commensals (Brugger et al., 2020).

For morphological characterization, colony morphology of *S. epidermidis* HK 95 was examined following growth on solid media. Gram staining was performed using standard protocols to assess cell morphology and Gram reaction (Gao et al., 2025). For ultrastructural analysis, bacterial cells were prepared for scanning electron microscopy (SEM). Briefly, cells were immersed in glutaraldehyde for fixation, processed through a stepwise ethanol dehydration series, coated with a thin layer of gold, and visualized using a scanning electron microscope to assess cell shape and surface morphology, as described previously (Li et al., 2022).

Growth dynamics of *S. epidermidis* HK 95 were evaluated in TSB by monitoring optical density at 600 nm (OD₆₀₀). Growth curves were generated to identify lag, exponential, and stationary growth phases according to established methods for bacterial growth analysis (Kwon et al., 2018).

For *in vitro* and *in vivo* experiments, bacterial cells were collected at mid-log phase, rinsed twice in sterile PBS, and resuspended at the indicated concentrations.

### 2.2 *In vitro* safety assessment

#### 2.2.1 Hemolytic activity assay of *Staphylococcus epidermidis* HK 95

The hemolytic activity of *S. epidermidis* HK 95 was assessed using a blood agar plate assay. Briefly, bacterial cultures were streaked onto blood agar plates containing 5% defibrinated sheep blood and incubated aerobically at 37 °C for 24–48 h. Hemolytic activity was evaluated by visually inspecting the zones surrounding bacterial colonies. *β*-hemolysis was identified by a clear, transparent zone indicating complete lysis of red blood cells. *α*-hemolysis was characterized by a greenish or partially discolored zone, indicating partial lysis. *γ*-hemolysis (non-hemolytic activity) was defined by the absence of any visible halo around the colonies. Isolates that exhibited no detectable hemolysis were classified as non-hemolytic. This assay followed established protocols for staphylococcal hemolysis evaluation (Elnar et al., 2025).

#### 2.2.2 Antibiotic susceptibility test

Antibiotic susceptibility was assessed by the agar disc diffusion assay following CLSI recommendations (Haley et al., 2024). Overnight cultures were normalized to a 0.5 McFarland turbidity and spread evenly onto Mueller–Hinton agar using a sterile swab.

Commercial antibiotic-impregnated discs (Oxoid, United Kingdom) were placed on the agar surface. The antibiotic panel included gentamicin (10 μg), amikacin (30 μg), vancomycin (30 μg), erythromycin (15 μg), amoxicillin–clavulanic acid (20/10 μg), penicillin (10 units), kanamycin (30 μg), tetracycline (30 μg), enrofloxacin (5 μg), ciprofloxacin (5 μg), trimethoprim–sulfamethoxazole (25 μg), and chloramphenicol (30 μg).

Plates were incubated at 37 °C under aerobic conditions for 18–24 h, after which inhibition halos were recorded in millimeters. Zone sizes were classified as susceptible (S), intermediate (I), or resistant (R) based on CLSI standards for Staphylococcus spp. Each assay was carried out in three independent repeats, with data presented as mean ± SD.

#### 2.2.3 Cell viability assay

Human bronchial epithelial BEAS-2B cells were maintained in Dulbecco’s Modified Eagle Medium/Nutrient Mixture F-12 (DMEM/F12; Gibco, United States) supplemented with 10% fetal bovine serum (FBS; Gibco, United States) and 1% penicillin–streptomycin (Gibco, United States), and cultured at 37 °C in a humidified incubator with 5% CO₂ (Coyle et al., 2023). Cell viability was assessed using the Cell Counting Kit-8 (Kim et al., 2025) according to the manufacturer’s instructions. Absorbance was measured at 450 nm using a microplate reader. Cell viability was expressed as a percentage relative to untreated control cells (Zhu et al., 2020). Experiments were conducted in no fewer than three independent replicates.

#### 2.2.4 Lactate dehydrogenase release assay

Cytotoxicity was further evaluated by measuring lactate dehydrogenase release into culture supernatants using a commercial LDH assay kit. LDH activity was quantified spectrophotometrically as an indicator of membrane integrity and cell damage (Yun et al., 2017). LDH release was expressed relative to untreated control cells, and maximum LDH release was determined using cells treated with lysis buffer. Experiments were conducted in no fewer than three independent replicates.

### 2.3 Genotypic safety screening

Genomic DNA was extracted from *S. epidermidis* HK 95 using a commercial bacterial genomic DNA extraction kit (TIANamp Bacteria DNA Kit, Tiangen, China) according to the manufacturer’s instructions, with a pre-lysis step to facilitate cell wall disruption. Briefly, bacterial pellets were resuspended in lysozyme-containing buffer and incubated at 37 °C for 30 min prior to DNA purification. The extracted genomic DNA was used as a template for PCR screening of genes encoding hemolysins, staphylococcal enterotoxins, biogenic amine–producing decarboxylases, and coagulase. Amplification was performed using gene-specific primers (Supplementary Table S1).

### 2.4 Genome sequencing and functional annotation

The whole-genome sequencing of *S. epidermidis* HK 95 was performed using the PacBio Sequel II platform (Pacific Biosciences, CA, United States) with HiFi circular consensus sequencing (CCS) technology. A SMRTbell library was constructed according to the manufacturer’s protocol. The quality and concentration of the library were quantified using a Qubit fluorometer (Thermo Fisher Scientific, United States), and the size distribution was validated using an Agilent 2,100 Bioanalyzer (Agilent Technologies, United States). Prior to sequencing, the library was complexed with sequencing primers and DNA polymerase using the PacBio Binding Kit (Pacific Biosciences, United States), followed by purification with AMPure PB Beads. Sequencing was executed on a PacBio Sequel II platform by Biomarker Technologies Corporation, Beijing, China.

Raw data were processed using SMRT Link software (v10.0) to generate high-quality HiFi reads. To ensure high-quality sequences for downstream analysis, reads shorter than 2,000 bp were filtered out. *De novo* genome assembly was performed using Hifiasm (v0.12). The resulting contigs were circularized and the replication origin was adjusted using Circlator (v1.5.5). To further enhance assembly precision, genome polishing was performed with Pilon (v1.22) using Illumina short-read data. These short reads underwent rigorous quality control, including adapter trimming and the removal of low-quality reads, prior to the polishing step.

Protein-coding genes were predicted using Prodigal (v2.6.3) and genome visualization was performed using Circos (v0.66). Functional annotation was conducted by aligning the predicted sequences against multiple databases, including NCBI non-redundant (NR), eggNOG/COG, Gene Ontology (GO), KEGG, and CAZyme (Wu et al., 2023; Wang et al., 2022). The complete genome sequence of *S. epidermidis* HK 95 has been deposited in the NCBI GenBank database under the accession number JBQSMF000000000.

### 2.5 *In vivo* airway function assays

#### 2.5.1 Animals

Animals were kept in temperature- and humidity-regulated rooms (25 ± 2 °C, 40–70% RH) under a 12-h light/dark regime, and were provided standard chow and water ad libitum. All experimental procedures complied with internationally endorsed guidelines on laboratory animal care and use. All mice used in this study were obtained from the Experimental Animal Center of Tongji Medical College, Huazhong University of Science and Technology (Wuhan, China). All mice experiments were approved by the Institutional Animal Care and Use Committee (IACUC) of Huazhong University of Science and Technology (approval no. 4317). All animal experiments involving guinea pigs were approved by the Animal Ethics Committee of the Food and Drug Safety Evaluation Center of the Hubei Provincial Academy of Preventive Medicine and the Hubei Provincial Center for Disease Control and Prevention (approval no. 202230212).

#### 2.5.2 Citric acid–induced cough assay

Cough responses were evaluated using a citric acid–induced cough model in mice and guinea pigs. Baseline cough frequency and latency to the first cough were recorded prior to treatment. Animals then received administration of *S. epidermidis* HK 95, *D. pigrum*, or the positive control compound codeine phosphate, followed by 0.4 M citric acid challenge, as previously described (Dong et al., 2020).

The cough responses were evaluated using both citric acid–induced guinea pig and mouse cough models, as previously described (Adner et al., 2020; Xu et al., 2018), with minor modifications. Guinea pigs were individually placed in a transparent exposure chamber (3 L) and challenged with an aerosolized 0.4 M citric acid solution delivered at an average pressure of 400 mmHg for 10 min. Cough latency and total cough count within 30 min after challenge were recorded. Only guinea pigs exhibiting a cough frequency between 8 and 30 during the initial challenge were selected for further evaluation. After 24 h, the selected guinea pigs were randomly assigned to experimental groups (*n* = 6 per group) and treated with saline (negative control), *S. epidermidis* HK 95 (2 × 10^9^ CFU in saline), *D. pigrum* (2 × 10^9^ CFU in saline), or codeine phosphate (positive control). A second citric acid challenge was performed 1 h post-administration, with cough latency and frequency recorded.

For the mouse model, mice were placed in a whole-body plethysmography chamber with a pressure transducer and nebulization port for aerosol delivery. After citric acid aerosol exposure, the latency to the first cough and the total number of coughs within 3 min were recorded. Mice exhibiting a cough frequency between 8 and 20 during the initial challenge were selected for subsequent experiments. After a 24 h recovery period, the selected mice were randomly assigned to experimental groups (*n* = 8 per group) and treated with saline, *S. epidermidis* HK 95 (2 × 10^8^ CFU in saline), *D. pigrum* (2 × 10^8^ CFU in saline), or codeine phosphate. A second citric acid challenge was performed one hour after administration, and the cough latency and frequency were compared before and after treatment to assess antitussive efficacy.

#### 2.5.3 Phenol red expectoration assay

The expectorant activity of *S. epidermidis* HK 95 was evaluated using a phenol red secretion assay, as previously described (Han et al., 2010). Mice were randomly assigned to experimental groups (*n* = 8 per group). Animals were treated with saline (negative control), *S. epidermidis* HK 95 (2 × 10^8^ CFU suspended in saline), *D. pigrum* (2 × 10^8^ CFU in saline), or guaifenesin as a positive control.

Thirty minutes after administration, mice were intraperitoneally injected with 5% (w/v) phenol red in physiological saline at a dose of 500 mg/kg. After an additional 30 min, animals were euthanized via CO₂ inhalation at 30–70% flow rate of chamber volume per minute in accordance with institutional animal care guidelines. The trachea from the thyroid cartilage to the main stem bronchi was carefully excised without damage. Each trachea was immediately immersed in 3 mL of 5% (w/v) sodium bicarbonate (NaHCO₃) solution and gently agitated for 30 min to extract the phenol red. The optical density of the resulting solution was measured at 557 nm using a spectrophotometer. The amount of phenol red was quantified based on a standard regression curve and used as an indicator of expectorant activity.

#### 2.5.4 Bronchoconstriction assay

The bronchodilating activity was evaluated using an acetylcholine chloride– and histamine–induced bronchoconstriction model in guinea pigs, as previously described (Adner et al., 2020). Guinea pigs were individually placed in a 3 L glass exposure chamber and challenged with an aerosolized mixture of 2% acetylcholine chloride and 0.1% histamine delivered at an average pressure of 400 mmHg for 12 s. The time interval from aerosol exposure to the onset of respiratory distress leading to loss of posture (preconvulsive time) was recorded.

Guinea pigs exhibiting a preconvulsive time between 30 and 90 s during the initial challenge were considered eligible for further experiments. After a 24 h recovery period, eligible animals were randomly assigned to experimental groups. Animals were treated with saline (negative control), *S. epidermidis* HK 95 (2 × 10^9^ CFU suspended in saline), *D. pigrum* (2 × 10^9^ CFU in saline), or aminophylline as a positive control. One hour after administration, guinea pigs were subjected to a second bronchoconstrictive challenge using the same protocol as described above. The preconvulsive time was recorded and used as an indicator of bronchodilating efficacy.

### 2.6 Acute toxicity study

The acute toxicity of *S. epidermidis* HK 95 was evaluated in mice. Mice received *S. epidermidis* HK 95 (2 × 10^8^ CFU suspended in sterile saline), while control animals received saline alone. Following administration, mice were continuously observed for 1 h and intermittently monitored for the subsequent 4 h for signs of acute toxicity, including changes in general behavior, locomotor activity, posture, and responsiveness. Mice were then observed daily for up to 14 days to assess delayed toxic effects or mortality.

At the end of the observation period, mice were fasted for 12 h. Blood samples were collected from the retro-orbital plexus under anesthesia with pentobarbital sodium (30 mg/kg, intraperitoneal injection) for hematological analysis. Whole blood was subjected to routine hematological examination to assess general systemic toxicity. Animals were euthanized via CO₂ inhalation at 30–70% flow rate of chamber volume per minute in accordance with institutional animal care guidelines. Major organs, including the liver, spleen, and kidneys, were carefully excised and weighed. Organ coefficients were calculated as the ratio of organ weight to body weight and expressed as a percentage.

### 2.7 Statistical analysis

All quantitative data are presented as mean ± standard deviation. Statistical analyses were performed using GraphPad Prism software (version 9.0, GraphPad Software, United States). For comparisons involving two groups, a two-tailed Student’s *t*-test was applied. For experiments involving two independent variables, two-way analysis of variance (two-way ANOVA) was performed. *p* values are indicated directly in the figures.

## 3 Results

### 3.1 Morphological and growth characteristics of the airway-derived isolate

**Figure 1:**
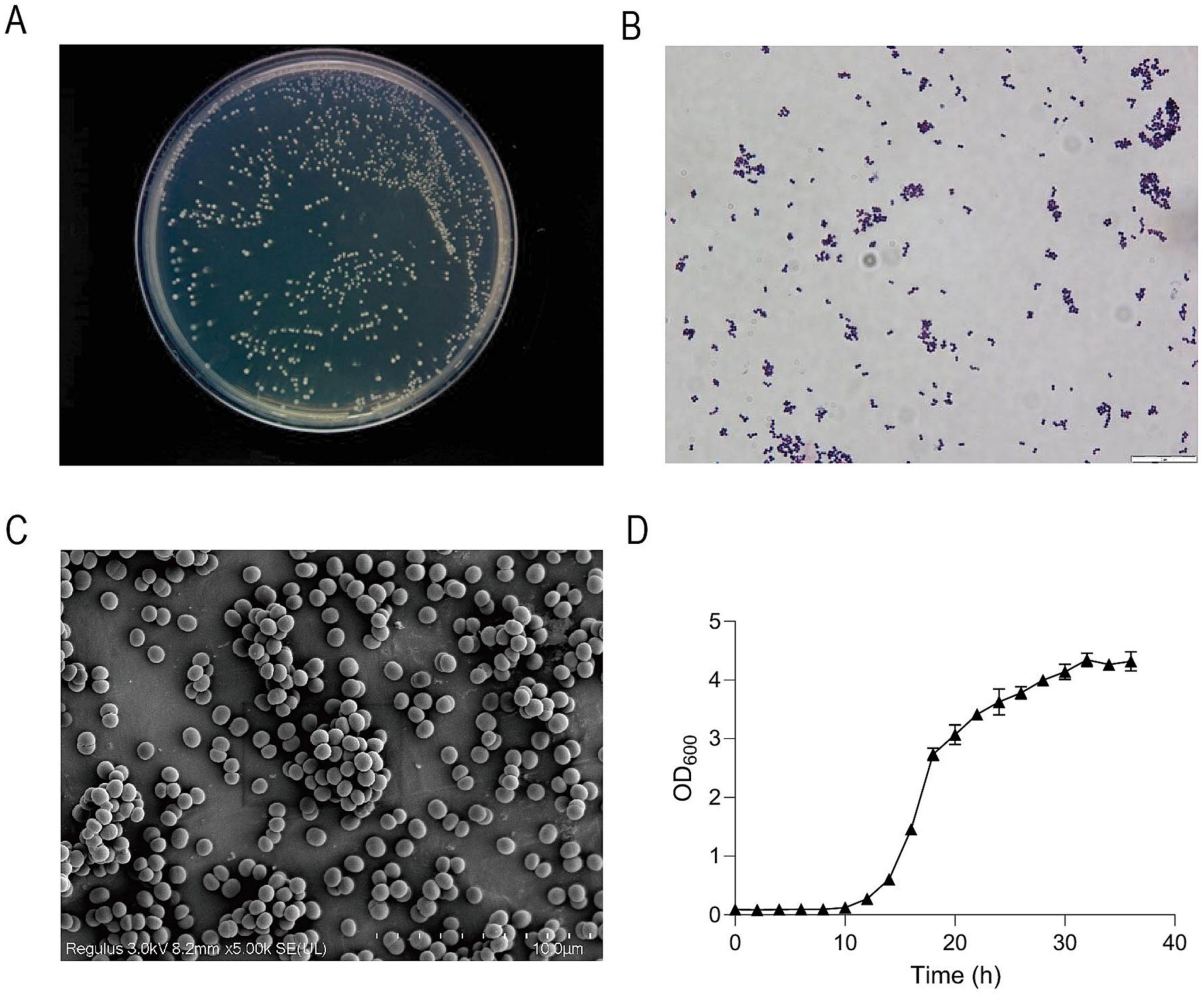
Morphological and growth characteristics of *S. epidermidis* HK 95. **(A)** Colony morphology of *S. epidermidis* HK 95 grown on solid medium. **(B)** Gram staining of *S. epidermidis* HK 95. **(C)** Scanning electron microscopy (SEM) image of *S. epidermidis* HK 95. **(D)** Growth curve of *S. epidermidis* HK 95 in liquid culture, monitored by measuring optical density at 600 nm (OD_600_) over time. Data in panel **(D)** are presented as mean ± SD.

An airway-derived bacterial isolate was initially obtained from nasal lavage fluid and cultured on solid medium. The isolate formed round, smooth, and opaque colonies with uniform size and well-defined margins (Figure 1A), consistent with typical staphylococcal colony morphology. Gram staining revealed Gram-positive cocci predominantly arranged in clusters (Figure 1B).

To confirm its taxonomic identity, 16S rRNA gene sequencing was performed, which verified that the isolate belongs to *S. epidermidis*. 16S rRNA gene sequence has been deposited in GenBank under accession number PV981942. Following this confirmation, the isolate was designated as *S. epidermidis* HK 95 for subsequent analyses.

Scanning electron microscopy (SEM) further revealed that *S. epidermidis* HK 95 cells were spherical with smooth surfaces and regular cell shapes, without apparent morphological abnormalities or surface appendages (Figure 1C). The growth dynamics of the isolate were then assessed in liquid culture. As shown in Figure 1D, *S. epidermidis* HK 95 exhibited a typical bacterial growth curve. A lag phase was observed during 0–10 h, followed by a rapid exponential growth phase between 10 and 22 h, and a stationary phase thereafter. This pattern is consistent with the known growth characteristics of *S. epidermidis* under nutrient-rich conditions, indicating good growth capacity and physiological stability of the isolate. These results demonstrate that the airway-derived isolate, *S. epidermidis* HK 95, displays canonical morphological features and growth characteristics consistent with commensal *S. epidermidis* strains.

### 3.2 Airway commensal *Staphylococcus epidermidis* HK 95 attenuates cough responses and enhances airway secretion in mice

**Figure 2:**
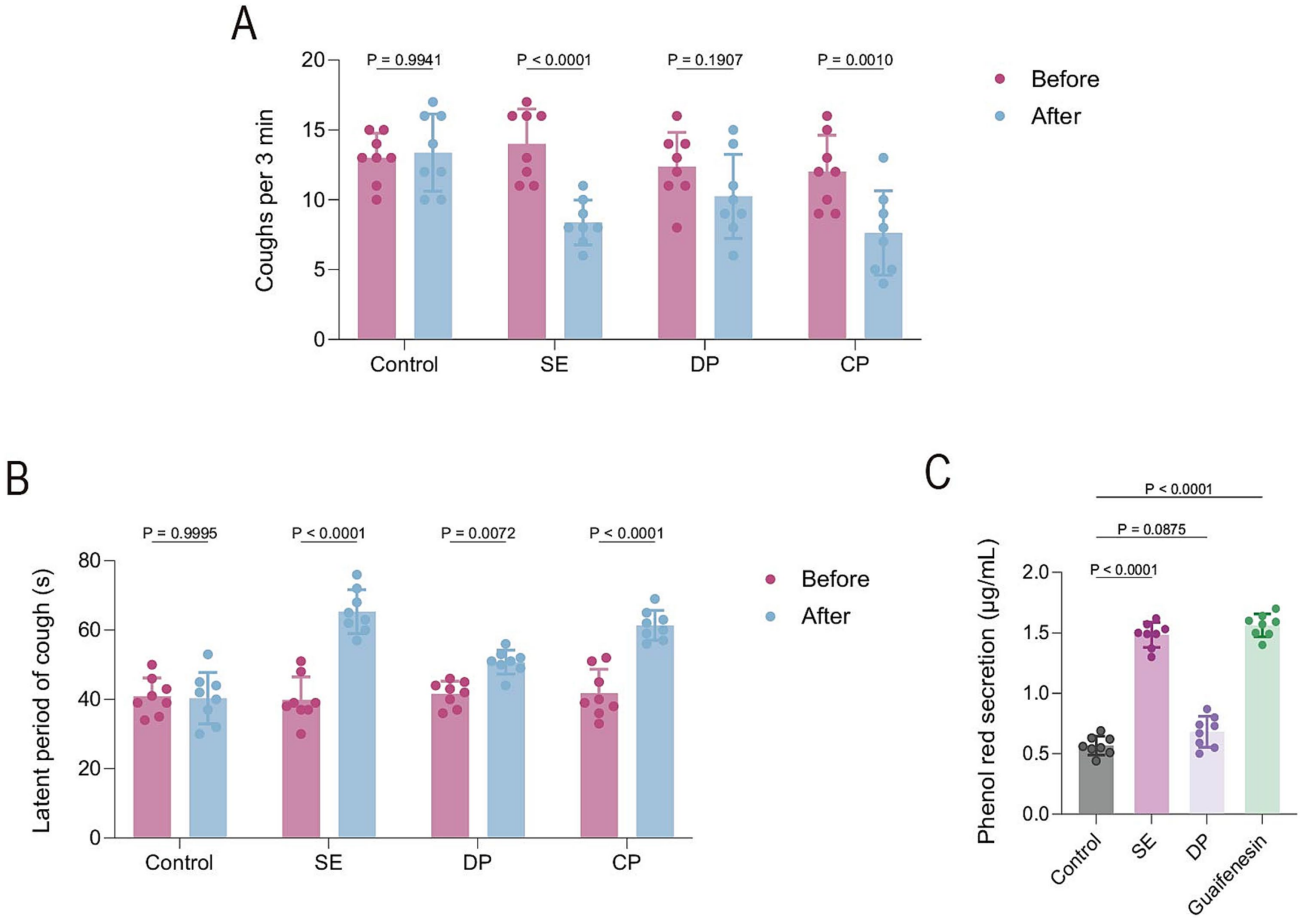
Effects of airway-derived *S. epidermidis* HK 95 on cough responses and airway secretion in mice. **(A)** Cough frequency (coughs per 3 min) in mice assessed using a citric acid–induced cough model. **(B)** Latent period to the first cough following citric acid challenge. **(C)** Airway secretion measured by the phenol red expectoration assay. *D. pigrum* represents a health-associated nasal commensal used as a comparator, codeine phosphate (CP) was used as a positive antitussive control, and guaifenesin was used as a reference expectorant in the phenol red assay. Data are presented as mean ± SD with individual data points shown. *p* values are indicated in the figure.

To evaluate the effects of airway-derived *S. epidermidis* HK 95 on airway function, cough responses were assessed using a citric acid–induced cough model in mice. As shown in Figure 2A, treatment with *S. epidermidis* HK 95 and *D. pigrum* reduced cough frequency within 3 min by 40% (*p* < 0.0001) and 17% (*p* = 0.1907), respectively, compared with pre-administration values. By comparison, treatment with the reference antitussive agent codeine phosphate resulted in a 36% (*p* = 0.001) reduction in cough frequency.

In addition to reducing cough frequency, *S. epidermidis* HK 95 markedly prolonged the latency to the first cough. *S. epidermidis* HK 95, codeine phosphate and *D. pigrum* significantly increased cough latency by 64% (*p* < 0.0001), 47% (*p* < 0.0001) and 22% (*p* = 0.0072), respectively, relative to baseline measurements (Figure 2B). These results indicate that *S. epidermidis* HK 95 effectively suppressed both the initiation and frequency of citric acid–induced cough in mice.

Airway secretory function was further evaluated using the phenol red expectoration assay. As shown in Figure 2C, administration of *S. epidermidis* HK 95 significantly increased tracheal phenol red secretion by approximately 1.6-fold compared with saline-treated controls (*p* < 0.0001). Treatment with the reference expectorant guaifenesin (50 mg/kg) resulted in a comparable 1.8-fold increase (*p* < 0.0001). In contrast, *D. pigrum* treatment induced a 20% increase in phenol red secretion that did not reach statistical significance (*p* = 0.0875). This enhancement of airway secretory function by *S. epidermidis* HK 95 likely contributes to improved mucociliary clearance, reflecting a broader capacity to support airway defense mechanisms beyond simply reducing cough.

### 3.3 Airway commensal *Staphylococcus epidermidis* HK 95 alleviates cough responses and bronchoconstriction in guinea pigs

**Figure 3:**
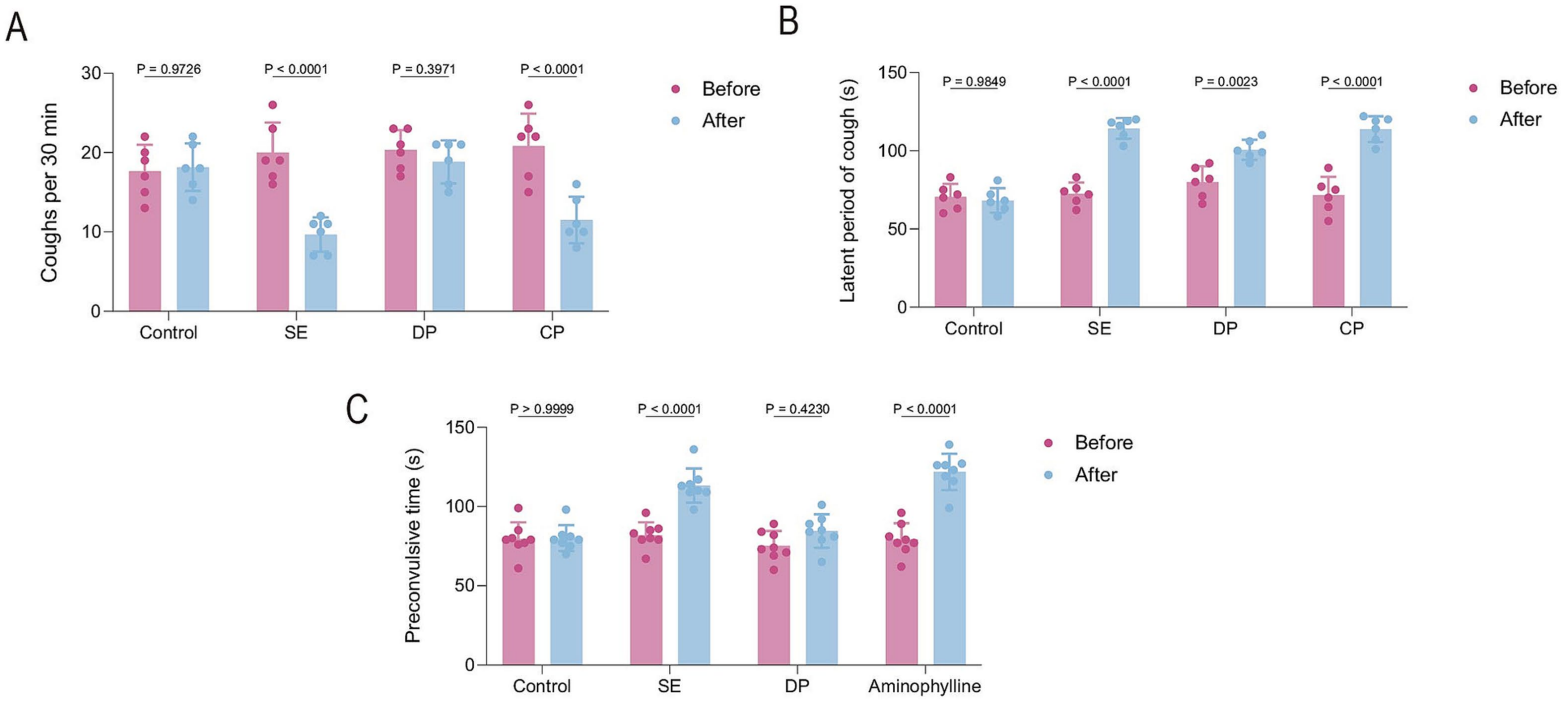
Effects of airway-derived *S. epidermidis* HK 95 on cough responses and bronchoconstriction in guinea pigs. **(A)** Cough frequency (coughs per 30 min) assessed using a citric acid–induced cough model. **(B)** Latent period to the first cough following citric acid challenge. **(C)** Preconvulsive time assessed in a bronchoconstriction model. Data are presented as mean ± SD with individual data points shown. *p* values are indicated in the figure.

To further validate the airway-protective effects of airway-derived *S. epidermidis* HK 95 across species, cough responses were evaluated using a citric acid–induced cough model in guinea pigs. As shown in Figure 3A, treatment with *S. epidermidis* HK 95 and *D. pigrum* reduced cough frequency within 30 min by 51% (*p* < 0.0001) and 7.3% (*p* = 0.397), respectively, compared with pre-administration values. Administration of the reference antitussive agent codeine phosphate led to a significant 45% reduction in cough frequency (*p* < 0.0001).

In addition to reducing cough frequency, *S. epidermidis* HK 95 markedly prolonged the latency to the first cough. As shown in Figure 3B, treatment with *S. epidermidis* HK 95 and *D. pigrum* increased cough latency by 58% (*p* < 0.0001) and 26% (*p* = 0.0023), respectively, relative to baseline measurements, whereas administration of codeine phosphate prolonged cough latency by 59% (*p* < 0.0001). These findings indicate that *S. epidermidis* HK 95 effectively suppresses both the initiation and frequency of citric acid–induced cough in guinea pigs.

To assess the effects on airway constriction, bronchoconstriction-related responses were examined using a preconvulsive time assay. As shown in Figure 3C, administration of the reference bronchodilator aminophylline markedly prolonged the preconvulsive time by approximately 54% compared with pre-administration values (*p* < 0.0001). Treatment with *S. epidermidis* HK 95 also significantly increased preconvulsive time by approximately 38% (*p* < 0.0001). In contrast, *D. pigrum* treatment induced a smaller increase of 12% (*p* = 0.4230). *S. epidermidis* HK 95 mitigates bronchoconstriction, reflecting a broader airway-protective role through modulation of airway smooth muscle reactivity and potential attenuation of airway irritation and inflammation.

### 3.4 Safety assessment *in vitro*

#### 3.4.1 Antibiotic susceptibility profile of *Staphylococcus epidermidis* HK 95

The antibiotic susceptibility profile of *S. epidermidis* HK 95 was evaluated using the agar disc diffusion method in accordance with CLSI guidelines. As summarized in Table 1, *S. epidermidis* HK 95 was susceptible to the majority of tested antibiotics, including amikacin, vancomycin, erythromycin, amoxicillin–clavulanic acid, kanamycin, tetracycline, enrofloxacin, trimethoprim–sulfamethoxazole and chloramphenicol. *S. epidermidis* HK 95 showed intermediate susceptibility to ciprofloxacin and displayed resistance only to gentamicin and penicillin, based on inhibition zone diameters (Table 1). The MAR value of *S. epidermidis* HK 95 was calculated as 0.17, reflecting resistance to only two out of the twelve agents tested. As this value remains below the commonly accepted threshold of 0.20, *S. epidermidis* HK 95 is unlikely to derive from settings subject to heavy antibiotic pressure and presents a low health risk in terms of acquiring or disseminating antibiotic resistance.

**Table 1:** Antibiotic susceptibility profile of *S. epidermidis* HK 95.

Antibiotic (abbreviation)	Inhibition zone (mm)	Interpretation
Gentamicin (CN)	7 ± 0.22	R
Amikacin (AK)	25 ± 0.32	S
Vancomycin (VA)	22 ± 0.25	S
Erythromycin (E)	28 ± 0.21	S
Amoxicillin–clavulanic acid (AMC)	30 ± 0.23	S
Penicillin (P)	14 ± 0.21	R
Kanamycin (KAN)	28 ± 0.21	S
Tetracycline (TET)	30 ± 0.31	S
Enrofloxacin (ENR)	25 ± 0.12	S
Ciprofloxacin (CIP)	18 ± 0.27	I
Trimethoprim–sulfamethoxazole (SXT)	31 ± 0.21	S
Chloramphenicol (C)	30 ± 0.24	S

Antibiotic susceptibility was assessed using the agar disc diffusion method. Diameters of inhibition zones were measured in millimeters and interpreted as susceptible (S) intermediate (I), or resistant (R) according to Clinical and Laboratory Standards Institute (CLSI) guidelines for Staphylococcus species. Values are presented as mean ± SD from independent experiments.

#### 3.4.2 *In vitro* cytotoxicity and hemolytic assessment of *Staphylococcus epidermidis* HK 95

**Figure 4:**
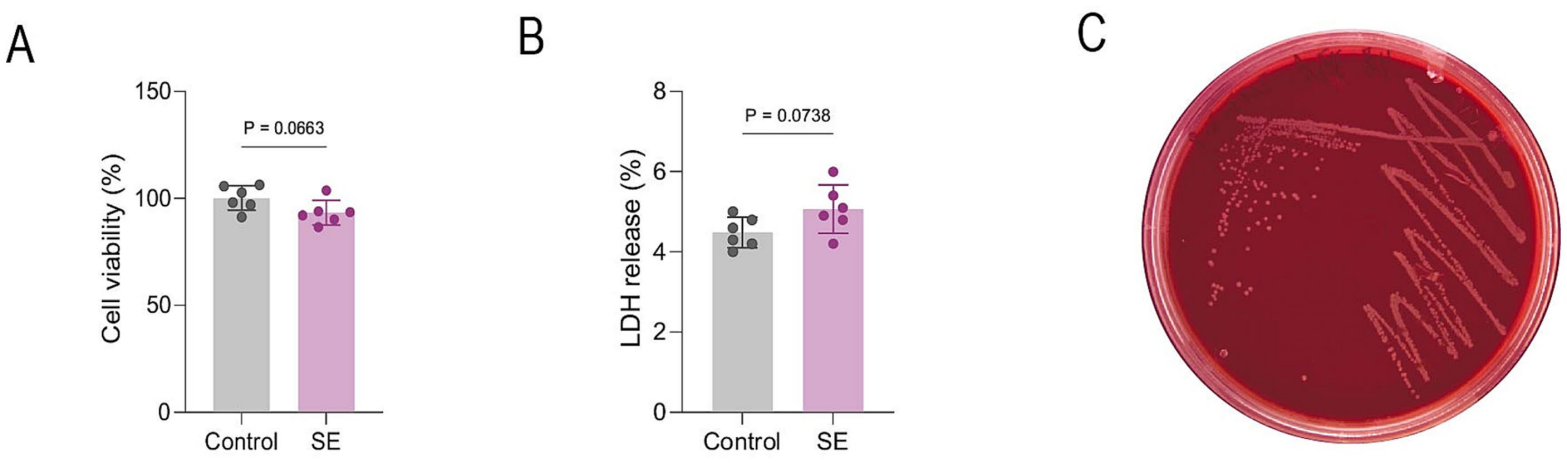
*In vitro* safety assessment of *S. epidermidis* HK 95. **(A)** Cell viability of BEAS-2B cells assessed by the CCK-8 assay following exposure to *S. epidermidis* HK 95. **(B)** Cytotoxicity evaluated by the lactate dehydrogenase (LDH) release assay. **(C)** Hemolytic activity of *S. epidermidis* HK 95 assessed on blood agar plates. Control indicates untreated cells or plates, and *S. epidermidis* HK 95 indicates treated groups. Data in panels **(A,B)** are presented as mean ± SD with individual data points shown. *p* values are indicated in the figure.

The *in vitro* safety of *S. epidermidis* HK 95 was further evaluated using human bronchial epithelial BEAS-2B cells and a hemolysis assay. Cell viability was first assessed by the CCK-8 assay following exposure to *S. epidermidis* HK 95. As shown in Figure 4A, treatment with *S. epidermidis* HK 95 did not significantly reduce BEAS-2B cell viability compared with the control group, and cell viability remained close to baseline levels, indicating the absence of overt cytotoxic effects.

Consistent with these findings, LDH release, a marker of membrane integrity and cell damage, was not significantly increased in *S. epidermidis* HK 95–treated cells compared with control cells (Figure 4B). LDH release levels in both groups remained low, further supporting the lack of detectable membrane damage induced by *S. epidermidis* HK 95.

In addition, the hemolytic activity of *S. epidermidis* HK 95 was examined on blood agar plates. No hemolytic zones were observed around *S. epidermidis* HK 95 colonies, indicating that the strain is non-hemolytic (Figure 4C). These results demonstrate that *S. epidermidis* HK 95 does not induce detectable cytotoxicity in airway epithelial cells and lacks hemolytic activity in vitro, supporting its favorable in vitro safety profile.

#### 3.4.3 Genotypic safety screening of *Staphylococcus epidermidis* HK 95

As summarized in Table 2, PCR-based screening did not detect genes encoding hemolysins, staphylococcal enterotoxins, biogenic amine–producing decarboxylases, or coagulase in *S. epidermidis* HK 95. Genes encoding staphylococcal enterotoxins and amino acid decarboxylases are responsible for the production of enterotoxins and biogenic amines, respectively, which are commonly associated with foodborne toxicity and adverse host responses. The absence of these genetic determinants therefore indicates a low toxigenic potential of *S. epidermidis* HK 95 at the genotypic level. Taken together with the phenotypic safety assessments described above, including the lack of hemolytic activity and cytotoxic effects, these genotypic screening results further support that *S. epidermidis* HK 95 is non-harmful to airway epithelial cells.

**Table 2:** PCR-based screening of virulence and toxin-associated genes in *S. epidermidis* HK 95.

Genes		Function notes	*S. epidermidis* HK 95
Hemolysin gene	*hla*	α-hemolysin	–
	*hlb*	*β-*hemolysin	–
	*hlg*	*γ-*hemolysin	–
	*hld*	*δ-*hemolysin	–
Enterotoxin gene	*sea*	Staphylococcal enterotoxin A	–
	*seb*	Staphylococcal enterotoxin B	–
	*sec*	Staphylococcal enterotoxin C	–
	*sed*	Staphylococcal enterotoxin D	–
	*see*	Staphylococcal enterotoxin E	–
	*seh*	Staphylococcal enterotoxin H	–
Decarboxylase gene	*hdc*	Histidine decarboxylase (histamine-producing)	–
	*tdc*	Tyrosine decarboxylase (tyramine)	–
	*odc*	Ornithine decarboxylase (putrescine)	–
	*ldc*	Lysine decarboxylase (cadaverine)	–
Coagulase gene	*coa*	Plasma-coagulase	–

### 3.5 *In vivo* safety evaluation of *Staphylococcus epidermidis* HK 95

**Figure 5:**
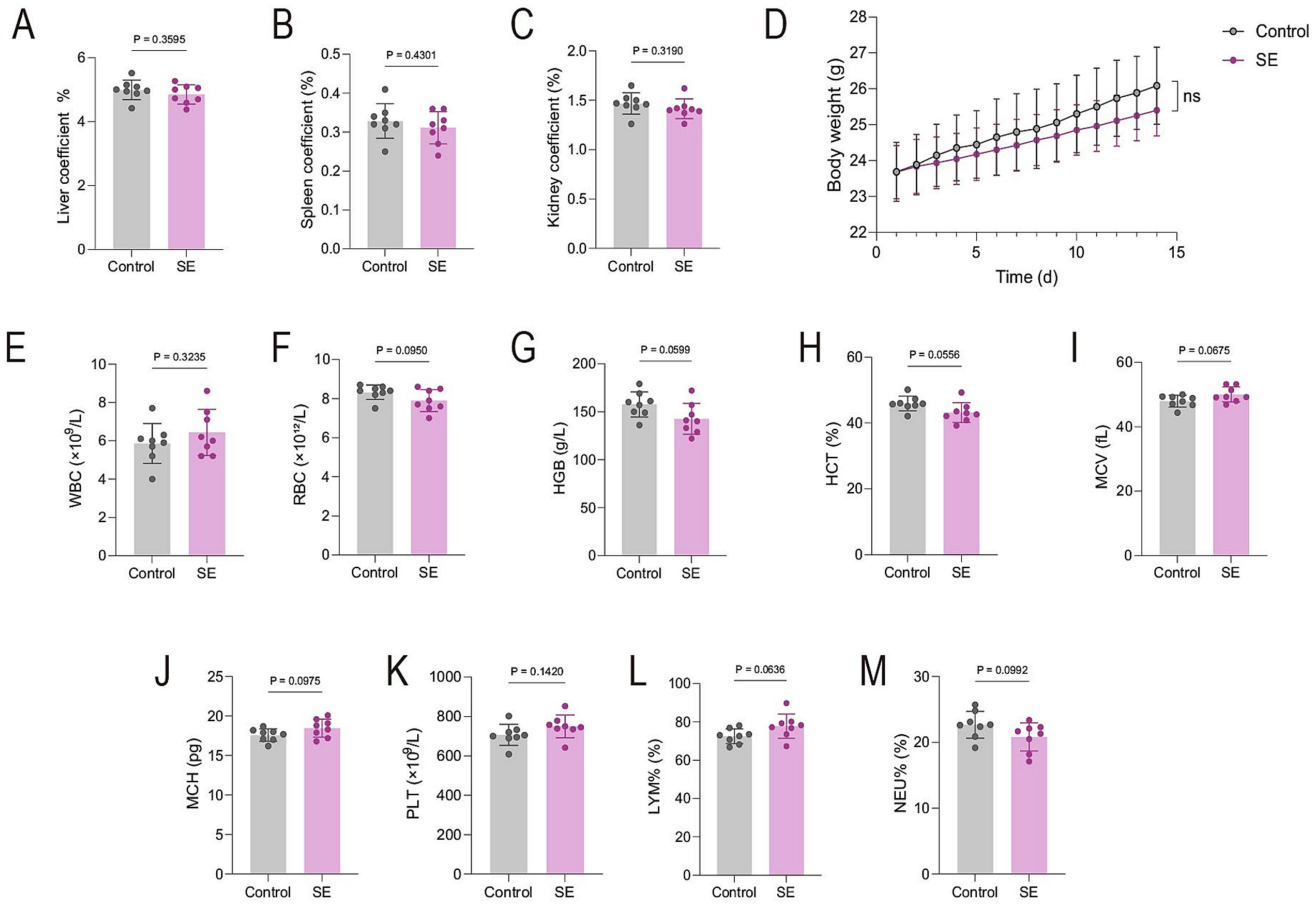
*In vivo* safety assessment of airway-derived *S. epidermidis* HK 95 in mice. **(A–C)** Liver, spleen, and kidney coefficients following repeated administration of *S. epidermidis* HK 95. **(D)** Body weight changes during the experimental period. **(E–M)** Hematological parameters, including white blood cell count (WBC), red blood cell count (RBC), hemoglobin (HGB), hematocrit (HCT), mean corpuscular volume (MCV), mean corpuscular hemoglobin (MCH), platelet count (PLT), lymphocyte percentage (LYM%), and neutrophil percentage (NEU%). Control indicates saline-treated mice, and *S. epidermidis* HK 95 indicates mice treated with *S. epidermidis* HK 95. Data are presented as mean ± SD with individual data points shown. Statistical significance was assessed as described in methods, and *p* values are indicated in the figure.

The *in vivo* safety of repeated administration of *S. epidermidis* HK 95 was evaluated in mice. Throughout the experimental period, all animals remained active, and no cases of diarrhea, mortality, or other overt clinical signs of illness were observed. Gross examination at necropsy revealed no apparent pathological alterations in major organs. Consistent with these observations, no significant differences were detected in body weight or organ coefficients, including liver, spleen, and kidney coefficients, between *S. epidermidis* HK 95–treated mice and saline-treated controls (Figures 5A–D; *p* > 0.05). Body weight was monitored throughout the experimental period as a general indicator of systemic health. As shown in Figure 5D, mice in the *S. epidermidis* HK 95 group exhibited a normal progressive increase in body weight over time, comparable to that observed in the control group, with no evidence of growth retardation or weight loss.

Hematological parameters were further analyzed to assess potential systemic or hematopoietic effects. No significant differences were observed between *S. epidermidis* HK 95–treated and control mice in white blood cell count, red blood cell count, hemoglobin concentration, hematocrit, mean corpuscular volume, mean corpuscular hemoglobin, platelet count, or leukocyte differential percentages, including lymphocytes and neutrophils (Figures 5E–M). These results indicate that repeated administration of *S. epidermidis* HK 95 does not induce overt systemic toxicity or hematological abnormalities in mice, underscoring its overall in vivo safety.

### 3.6 Genomic characterization of *Staphylococcus epidermidis* HK 95

**Figure 6:**
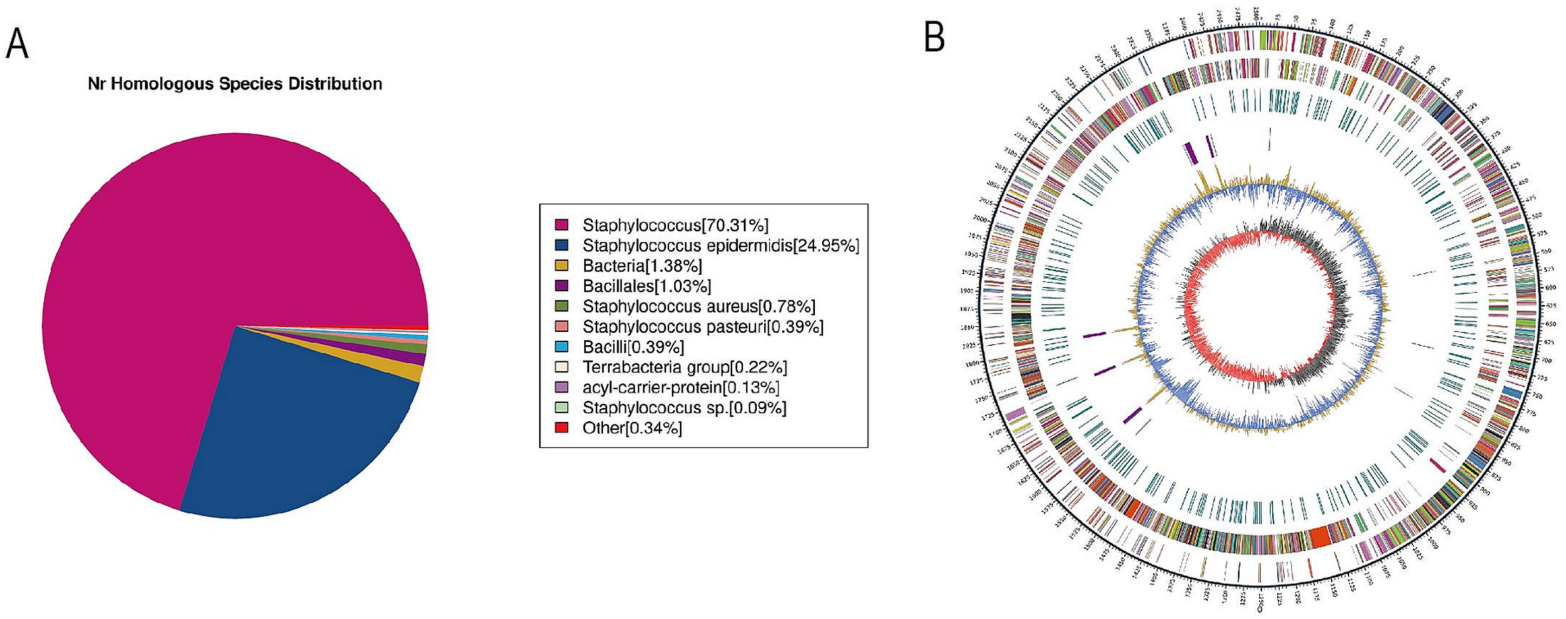
Genomic characterization of *S. epidermidis* HK 95. **(A)** Species distribution of homologous proteins based on BLASTP analysis against the NCBI non-redundant (NR) database. **(B)** Circular representation of the complete genome of *S. epidermidis* HK 95. From the outermost to the innermost rings: (1) Genome scale indicated at 5 kb intervals; (2) coding sequences (CDSs) on the forward strand; (3) CDSs on the reverse strand (both color-coded according to COG functional categories); (4) repeat sequences; (5) tRNA (blue) and rRNA (purple) genes; (6) GC content (light yellow: above average, blue: below average); and (7) GC skew (dark grey: G > C, red: C > G).

The genome of *S. epidermidis* HK 95 was sequenced using PacBio HiFi long-read sequencing and taxonomically classified as *S. epidermidis*. High-coverage sequencing data were generated, resulting in a complete genome assembly of 2.57 Mb with an average GC content of 32.04%. Comparative analysis of predicted genes based on BLAST annotations revealed that the majority of sequences exhibited high homology to *S. epidermidis*, supporting species-level classification (Figure 6A). Genome annotation predicted a total of 2,341 genes, including protein-coding sequences as well as ribosomal RNA and transfer RNA genes. A circular representation of the complete genome, illustrating gene distribution, GC content, and other genomic features, is shown in Figure 6B. The complete genome sequence of *S. epidermidis* HK 95 has been deposited in the NCBI under accession number JBQSMF000000000.

### 3.7 Functional gene annotation of *Staphylococcus epidermidis* HK 95

**Figure 7:**
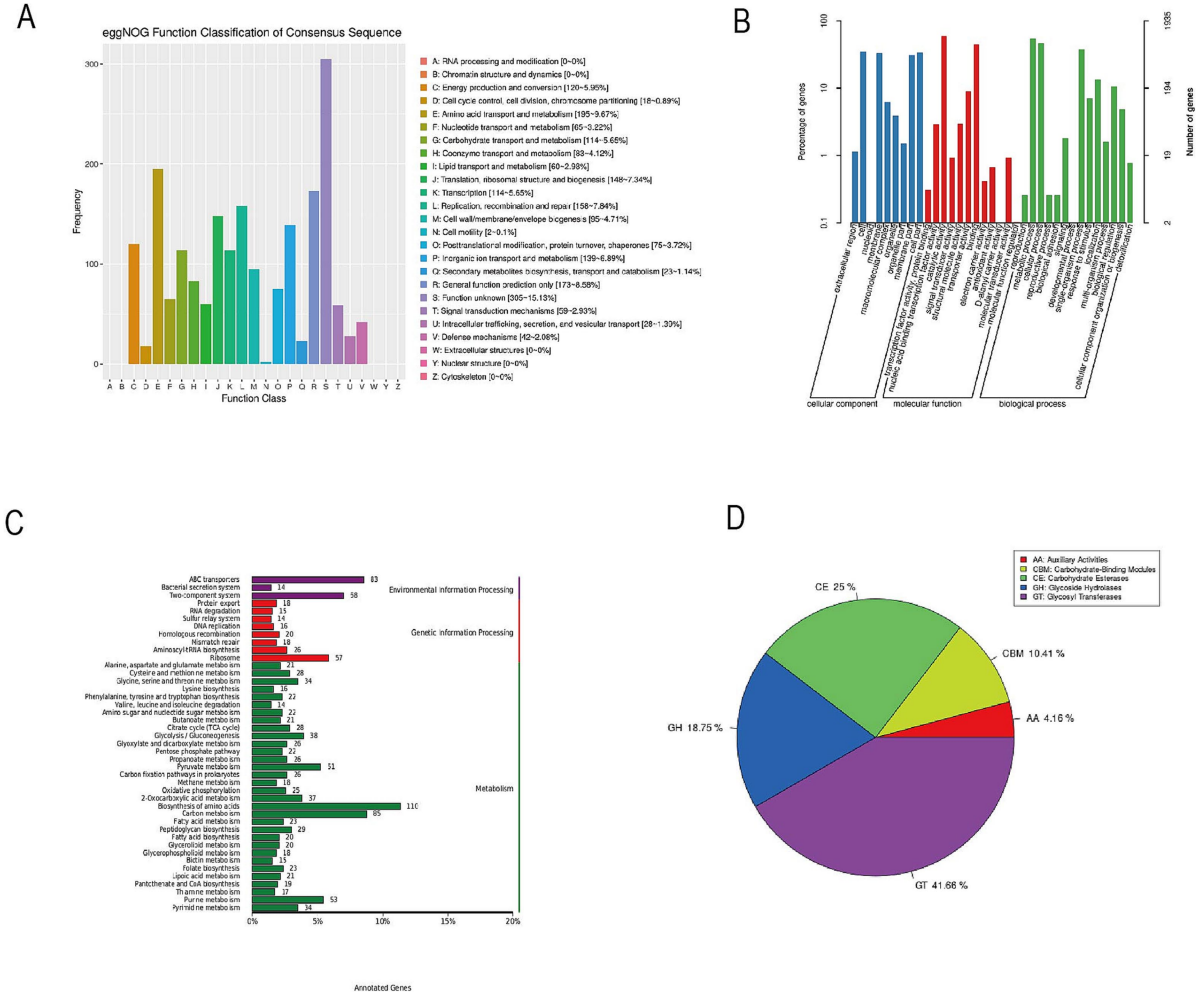
Genome-wide functional annotation of *S. epidermidis* HK 95. **(A)** EggNOG/COG functional classification of predicted proteins. **(B)** Gene Ontology (GO) functional classification. **(C)** KEGG pathway annotation. **(D)** CAZyme functional classification.

To obtain a comprehensive overview of the functional potential of *S. epidermidis* HK 95, genome-wide functional annotation was performed using the eggNOG/COG, Gene Ontology (GO), KEGG pathway, and CAZyme databases. As shown in Figure 7A, eggNOG/COG functional annotation revealed that protein-coding genes were distributed across a broad range of functional categories. A substantial proportion were assigned to core cellular processes, including translation and ribosomal structure (J), energy production and conversion (C), transcription (K), replication and repair (L), and cell wall/membrane biogenesis (M). In addition, a notable number of genes fell into the “general function prediction only” (R) and “function unknown” (S) categories.

Consistent with these observations, GO-based functional annotation indicated that genes were broadly distributed across biological processes, molecular functions, and cellular components. The majority of biological process terms mapped to cellular and metabolic processes, while molecular function annotations were dominated by catalytic activity and binding. Cellular component categories were enriched in membrane-associated and intracellular structures (Figure 7B). KEGG pathway analysis further confirmed that most annotated genes participated in metabolic pathways, including carbohydrate, amino acid, lipid, and nucleotide metabolism, along with transport systems and genetic information processing pathways such as replication, transcription, and translation (Figure 7C). In addition, CAZyme profiling revealed that glycosyltransferases represented the most abundant class of carbohydrate-active enzymes, followed by carbohydrate esterases and glycoside hydrolases (Figure 7D), consistent with a balanced capacity for carbohydrate modification, degradation, and biosynthesis. These functional annotation results indicate that *S. epidermidis* HK 95 possesses a complete and well-balanced functional repertoire characteristic of a commensal *S. epidermidis* strain, supporting its metabolic competence and adaptation to the airway environment.

## 4 Discussion

The airway microbiota is increasingly recognized as an important contributor to respiratory health; however, the functional roles of individual airway commensal bacteria remain insufficiently characterized. Rather than acting merely as compositional correlates, airway commensals are emerging as functional modulators of airway physiology and immune balance (Natalini et al., 2023; Perdijk et al., 2024). In this study, we systematically characterized an airway commensal *S. epidermidis* HK 95, and demonstrated its airway-protective effects together with a favorable safety profile. By integrating *in vitro* safety assessments, *in vivo* functional models across two animal species, and comprehensive genomic analyses, our findings extend current understanding of airway commensals beyond descriptive ecological observations and support a functional role for *S. epidermidis* in the regulation of airway physiological responses.

Using a citric acid–induced acute cough model, we observed that administration of *S. epidermidis* HK 95 significantly reduced cough frequency and prolonged cough latency in both mice and guinea pigs. The consistency of these effects across species supports the robustness of our observations and indicates that airway commensal *S. epidermidis* can modulate airway sensory responsiveness in vivo. Citric acid–induced cough is commonly employed to model chemically evoked airway irritation and activation of vagal afferent pathways, which are central to acute airway sensory signaling (Canning et al., 2014; Jakusova and Pecova, 2022; Li et al., 2025). The attenuation of cough responses following *S. epidermidis* HK 95 administration therefore suggests a capacity of this commensal bacterium to dampen acute airway sensory hyperresponsiveness, reflecting a modulation of airway responsiveness under irritant challenge.

Beyond its effects on sensory responsiveness, *S. epidermidis* HK 95 significantly enhanced airway secretion, as assessed by the phenol red expectoration assay. Adequate airway secretion is critical for mucociliary clearance and the maintenance of airway homeostasis, and impaired secretion is a common feature of several airway disorders (Hill et al., 2022; Roth et al., 2025). The concurrent modulation of airway sensory responsiveness and enhancement of airway secretion suggest that *S. epidermidis* HK 95 contributes to restoring overall airway physiological balance rather than exerting an isolated symptomatic effect.

In addition to modulating airway sensory responses and secretion, *S. epidermidis* HK 95 alleviated bronchoconstriction in guinea pigs, as evidenced by prolonged preconvulsive time. Bronchoconstriction reflects heightened airway smooth muscle responsiveness and is closely associated with airway irritation and inflammation (Calzetta et al., 2022; Camoretti-Mercado and Lockey, 2021). The comparable efficacy observed between *S. epidermidis* HK 95 and aminophylline, a clinically established bronchodilator, suggests that airway commensal bacteria may influence pathways regulating airway tone and responsiveness.

While the precise molecular mechanisms underlying these effects remain to be defined, accumulating evidence indicates that airway commensals can shape epithelial function, immune signaling, and neuronal pathways within the airway (Cuthbertson et al., 2024; Kayongo et al., 2022; Mann-Nüttel et al., 2025). Our findings are consistent with this emerging concept and support a model in which commensal microbes actively participate in maintaining airway functional homeostasis rather than acting as passive colonizers.

A favorable safety profile is a prerequisite for any commensal-based intervention targeting the airway. *S. epidermidis* HK 95 exhibited no detectable cytotoxicity toward human bronchial epithelial cells and did not induce LDH release or hemolysis *in vitro*, consistent with the generally low virulence of commensal *S. epidermidis* strains compared with pathogenic staphylococci (Burke et al., 2024; Nurxat et al., 2023). Antibiotic susceptibility testing further demonstrated sensitivity to multiple clinically relevant antibiotics, indicating a low risk of multidrug resistance.

Genotypic screening corroborated these phenotypic observations, revealing the absence of genes encoding hemolysins, enterotoxins, biogenic amine–producing decarboxylases, and coagulase—key virulence determinants in pathogenic staphylococci (Bojang et al., 2024; Lee et al., 2024; Santos et al., 2025). *In vivo* safety evaluation further supported these findings, as repeated administration of *S. epidermidis* HK 95 did not result in overt clinical abnormalities, significant changes in body weight, hematological disturbances, or alterations in organ coefficients. These results support the in vitro and *in vivo* safety of *S. epidermidis* HK 95 under the tested conditions.

Whole-genome sequencing and functional annotation revealed that *S. epidermidis* HK 95 possesses a genomic architecture characteristic of commensal *S. epidermidis* strains, with enrichment in core metabolic functions, genetic information processing, and cell wall and membrane biogenesis. The predominance of carbohydrate-active enzymes, including glycosyltransferases and glycoside hydrolases, suggests metabolic flexibility that may facilitate adaptation to the nutrient-limited and dynamic airway environment. Importantly, no canonical pathogenicity-associated genes were identified, in agreement with the phenotypic and *in vivo* safety assessments. These genomic characteristics align with previous descriptions of *S. epidermidis* as a versatile commensal capable of interacting with host tissues while maintaining a low virulence profile (Park et al., 2019; Su et al., 2020).

Recent studies have increasingly emphasized the importance of the airway microbiota in respiratory health, with health-associated nasal communities often enriched in commensal taxa such as *D. pigrum* and *Corynebacterium* spp. (Dickson et al., 2014b; Drigot and Clark, 2024; Du et al., 2025; Yu et al., 2025). However, much of the existing literature has focused on compositional associations rather than direct functional outcomes. Our study advances this field by providing functional evidence that an airway commensal bacterium, *S. epidermidis* HK 95, can actively modulate airway physiological responses in vivo. While *D. pigrum* has frequently been regarded as a marker of a healthy airway microbiota, functional studies directly linking airway commensals to modulation of airway function remain limited. In this context, our findings identify *S. epidermidis* as an active functional modulator of airway physiology rather than a passive indicator of microbial community composition.

Several limitations of this study should be acknowledged. First, although robust functional effects were observed, the underlying molecular mechanisms remain undefined. Future studies should explore how *S. epidermidis* HK 95 influences airway epithelial signaling, immune mediators, or sensory neuronal pathways that collectively shape airway responsiveness. In addition, the impact of *S. epidermidis* HK 95 on disease-specific airway pathology, mucus composition, epithelial barrier integrity, and inflammatory responses should be evaluated in relevant respiratory disease models to provide a more comprehensive understanding of its functional effects. Second, as the present findings are based on animal models, translation to human airway physiology will require careful clinical validation. Finally, long-term colonization dynamics and interactions with the native airway microbiota were not addressed and warrant further investigation, consistent with emerging perspectives on the complexity of lung microbiome–host interactions.

## 5 Conclusion

In conclusion, our findings demonstrate that airway-derived *S. epidermidis* HK 95 exerts antitussive, expectorant, and bronchoprotective effects *in vivo* while maintaining a favorable safety profile. These results provide functional support for the emerging concept that airway commensal bacteria contribute actively to respiratory homeostasis and highlight *S. epidermidis* HK 95 as a promising candidate for future microbiota-based strategies aimed at modulating airway function.

## Data Availability

The whole-genome sequence data of *Staphylococcus epidermidis* HK 95 presented in the study are deposited in the NCBI GenBank repository, accession number JBQSMF000000000.
