# Supplementary material for: Functional characterization and safety evaluation of an airway commensal Staphylococcus epidermidis HK 95
Source: Front Microbiol. 2026 Jun 17;17:1787099. doi: 10.3389/fmicb.2026.1787099 (PMC13319099; doi:10.3389/fmicb.2026.1787099)
Supplement: Supplementary file 1 [file Table_1.docx]

**Supplementary Table S1| PCR primers used to detect hemolysin, staphylococcal enterotoxin, biogenic amine–producing decarboxylase, and coagulase genes.**

| **Genes** | **Function notes** | | **Sequence** | **References** |
| --- | --- | --- | --- | --- |
| Hemolysin gene | *hla* | α-hemolysin | *HLA-F*:CTG ATT ACT ATC CAA GAA ATT CGA TTG | (Jarraud et al., 2002) |
|  |  |  | *HLA-R*:CTT TCC AGC CTA CTT TTT TAT CAG T |  |
|  | *hlb* | *β-*hemolysin | *HLB-F*:GTG CAC TTA CTG ACA ATA GTG C | (Jarraud et al, 2002) |
|  |  |  | *HLB-R*:GTT GAT GAG TAG CTA CCT TCA GT |  |
|  | *hlg* | *γ-*hemolysin | *mpHLG-F*:GTC AYA GAG TCC ATA ATG CAT TTA A | (Jarraud et al, 2002) |
|  |  |  | *mpHLG-R*:CAC CAA ATG TAT AGC CTA AAG TG |  |
|  | *hld* | *δ-*hemolysin | *HLD-F*:AAG AAT TTT TAT CTT AAT TAA GGA AGG AGT G | (Jarraud et al, 2002) |
|  |  |  | *HLD-R*:*TTA GTG AAT TTG TTC ACT GTG TCG A* |  |
| Enterotoxin gene | *sea* | *Staphylococcal enterotoxin* A | SEA-F:*CCT TTG GAA ACG GTT AAA ACG* | (Kanjan and Sakpetch, 2020) |
|  |  |  | SEA-R:*CTG AAC CTT CCC ATC AAA AAC* |  |
|  | *seb* | *Staphylococcal enterotoxin* B | SEB-F:*GGT ACT CTA TAA GTG CCT GC* | (Kanjan and Sakpetch, 2020) |
|  |  |  | SEB-R:*TTC GCA TCA AAC TGA CAA ACG* |  |
|  | *sec* | *Staphylococcal enterotoxin* C | SEC-F:*AGA ACT AGA CAT AAA AGC TAG G* | (Kanjan and Sakpetch, 2020) |
|  |  |  | SEC-R:*TCA AAA TCG GAT TAA CAT TAT CC* |  |
|  | *sed* | *Staphylococcal enterotoxin* D | SED-F:*TTT GGT AAT ATC TCC TTT AAA CG* | (Kanjan and Sakpetch, 2020) |
|  |  |  | SED-R:*CTA TAT CTT ATA GGG TAA ACA TC* |  |
|  | *see* | *Staphylococcal enterotoxin* E | SEE-F:*CCT ATA GAT AAA GTT AAA ACA AGC* | (Kanjan and Sakpetch, 2020) |
|  |  |  | SEE-R:*TAA CTT ACC GTG GAC CCT TC* |  |
|  | *seh* | *Staphylococcal enterotoxin* H | SEH-F:*CAA TCA CAT CAT ATG CGA AAG CAG* | (Müller et al., 2016) |
|  |  |  | SEH-R:*CAT CTA CCC AAA CAT TAG CAC C* |  |
| Decarboxylase gene | *hdc* | Histidine decarboxylase (histamine-producing) | HIS1-F:GGN ATN GTN WSN TAY GAY MGN GCN GA | (de las Rivas et al., 2006) |
|  |  |  | HIS1-R:ATN GCD ATN GCN SWC CAN ACN CCR TA |  |
|  | *tdc* | Tyrosine decarboxylase (tyramine) | TDC-F:TGG YTN GTN CCN CAR CAN AAR CAY TA | (de las Rivas et al, 2006) |
|  |  |  | TDC-R:ACR TAR TCN ACC ATR TTR AAR TCN GG |  |
|  | *odc* | Ornithine decarboxylase (putrescine) | PUT1-F:TWY MAY GCN GAY AAR CAN TAY YYT GT | (de las Rivas et al, 2006) |
|  |  |  | PUT1-R:ACR CAN AGN CAN CCN GGN GGR TAN GG |  |
|  | *ldc* | Lysine decarboxylase (cadaverine) | CAD2-F:CAY RTN CCN GGN CAY AA | (de las Rivas et al, 2006) |
|  |  |  | CAD2-R:GGD ATN CCN GGN GGR TA |  |
| Coagulase gene | *coa* | Plasma-coagulase | COA-F:ATA GAG ATG CTG GTA CAG G | (Moura et al., 2012) |
|  |  |  | COA-R:GCT TCC GAT TGT TCG ATG C |  |

**References**

de las Rivas, B., Marcobal, A., Carrascosa, A. V. and Muñoz, R. (2006). PCR detection of foodborne bacteria producing the biogenic amines histamine, tyramine, putrescine, and cadaverine. *J. Food Prot.* 69, 2509–2514. doi: 10.4315/0362-028x-69.10.2509

Jarraud, S. *et al.* (2002). Relationships between *Staphylococcus aureus* genetic background, virulence factors, agr groups (alleles), and human disease. *Infect. Immun.* 70, 631–641. doi: 10.1128/iai.70.2.631-641.2002

Kanjan, P. and Sakpetch, P. (2020). Functional and safety assessment of *Staphylococcus simulans* PMRS35 with high lipase activity isolated from high salt-fermented fish (Budu) for starter development. *LWT* 124. doi: 10.1016/j.lwt.2020.109183

Moura, T. M. *et al.* (2012). Prevalence of enterotoxin-encoding genes and antimicrobial resistance in coagulase-negative and coagulase-positive Staphylococcus isolates from black pudding. *Rev. Soc. Bras. Med. Trop.* 45, 579–585. doi: 10.1590/s0037-86822012000500008

Müller, A. *et al.* (2016). Safety assessment of selected *Staphylococcus carnosus* strains with regard to their application as meat starter culture. *Food Control* 66, 93–99. doi: 10.1016/j.foodcont.2016.01.042
